# Address of Dr. T. A. Goodwin, President of the Northern Indiana Dental Society

**Published:** 1900-12-15

**Authors:** 


### ADDRESS OF DR. T. A. GOODWIN, PRESIDENT OF THE NORTHERN INDIANA DENTAL SOCIETY.

---

*Mr. President, and Members of the Southwestern Michigan Dental Association:*—In behalf of the Association which I represent, I most cordially accept the hearty welcome you have extended.

We have assembled here to hold the regular meeting of the Northern Indiana Dental Society, in connection with the Southwestern Michigan Dental Association; both associations have for their objects the mutual benefit of their members and the honor of our profession. These unions are increasing and will unquestionably aid in raising dentistry to a higher place among the professions, for "In union there is strength."

We have met here, then, fellow-practitioners, for that mutual exchange of thoughts, ideas and experiences which will aid us in attaining the goal for which we are all striving, namely, consummate excellence.

What changes have been wrought in the past century by the inventive skill of man; in no field has this change been more marked or more rapid than in dentistry. There are few professions whose origin has been so recent as to leave us any knowledge concerning their beginning. This is not the case in ours, however. During the sixteenth century the first dissertations upon the anatomy and treatment of the teeth were given to the public.

The seventeenth century was more favored, while the eighteenth produced some masterpieces frcm the pens of progressive thinkers.

Until the latter part of the eighteenth century dentistry was confined to Europe, being introduced to our own country during the Revolutionary struggle.

The great progress made by the dental profession has been contributed, and stimulated not a little, by the organization of colleges, dental societies and the publication of books and proceedings of dental societies in the dental journals.

An important event in the history of dental surgery in this country was the formation of the American Society of Dental Surgeons in 1840, the first of the kind in America. Two years later another society was formed at Richmond, Va., and in 1844 a third was formed at Cincinnati, Ohio. In 1850 the National Society was organized, and in 1863 the Odontolographic Society, of Philadelphia.

To-day, in addition to these, there are throughout the country a large number of State and district societies.

The law of the universe is that of progress. A growing desire on the part of liberal and educated men in the profession that their specialty should be raised above the mere mechanical trade has created a necessity for some medium through which this may be accomplished, this is being done to a great extent through the dental colleges; to a great extent, I say, and yet a college education serves only as a foundation for that knowledge which each one who honors his profession must acquire.

Let me say to the younger members of our profession and to those who are still students, if any such be present, be eager for the facts that belong to the substructure rather than those that belong to the finish of culture. The deeper you go into principles, the higher you will rise in results in the years to come. Would that you might account those years as precious to you, as those who are engaged in the wear and waste of professional toil know and feel they are.

Concentrated power and untiring research should bring to the laborer a sweet, mental satisfaction.

The Northern Indiana Dental Society is a subdivision of the State Association. Therefore its name and object is materially the same as the former, yet I consider that we individually reap more benefit from being members of the district association.

This is a self-evident fact, because we have more time to deal with the practical and minor points, while the State Society must, of necessity, develop and dwell upon the more scientific topics. In order that we may be worthy members of a society we must perform the parts allotted to us to the best of our ability, and realize that upon each of us as well as upon the society, as a body, rests the responsibility of elevating the standard of dentistry.

